# A Multidimensional IRT Approach for Dynamically Monitoring Ability Growth in Computerized Practice Environments

**DOI:** 10.3389/fpsyg.2019.00620

**Published:** 2019-03-29

**Authors:** Jung Yeon Park, Frederik Cornillie, Han L. J. van der Maas, Wim Van Den Noortgate

**Affiliations:** ^1^Faculty of Psychology and Educational Sciences, imec–ITEC, KU Leuven, Leuven, Belgium; ^2^Department of Psychology, University of Amsterdam, Amsterdam, Netherlands

**Keywords:** multidimensional IRT, Elo rating system, adaptive practice, speed-accuracy trade-off, e-learning

## Abstract

Adaptive learning systems have received an increasing attention as they enable to provide personalized instructions tailored to the behaviors and needs of individual learners. In order to reach this goal, it is desired to have an assessment system, monitoring each learner's ability change in real time. The Elo Rating System (ERS), a popular scoring algorithm for paired competitions, has recently been considered as a fast and flexible method that can assess learning progress in online learning environments. However, it has been argued that a standard ERS may be problematic due to the multidimensional nature of the abilities embedded in learning materials. In order to handle this issue, we propose a system that incorporates a multidimensional item response theory model (MIRT) in the ERS. The basic idea is that instead of updating a single ability parameter from the Rasch model, our method allows a simultaneous update of multiple ability parameters based on a compensatory MIRT model, resulting in a multidimensional extension of the ERS (“M-ERS”). To evaluate the approach, three simulation studies were conducted. Results suggest that the ERS that incorrectly assumes unidimensionality has a seriously lower prediction accuracy compared to the M-ERS. Accounting for both speed and accuracy in M-ERS is shown to perform better than using accuracy data only. An application further illustrates the method using real-life data from a popular educational platform for exercising math skills.

## Introduction

Over the past decade adaptive learning systems have received an increasing attention as they enable to provide instructions tailored to the behaviors, needs, and learning pace of individual learners. In this way the learners can benefit from more personalized learning items. Therefore, it is desired for the systems to have a learner modeling method that keeps track of the learner's cognitive states and its evolution in a timely and flexible manner. In the context of computerized adaptive testing (CAT; van der Linden, 2000) the use of item response theory (IRT) is a common method to model the relationship between the learner's ability level and their responses to different measurable items.

As originally intended for high-stakes standardized tests, studies related to CAT primarily zooms in on how to increase the precision of the examinee's ability level estimate by successively rendering most informative items, or how to decrease the number of test items while maintaining a high level of precision in estimating the ability level. Because typically no feedback is given during the test, the true ability level is not expected to evolve. The idea of CAT also can be applied to learning environments in which learners interact with items, toward computerized adaptive practice (CAP; Klinkenberg et al., 2011). An a-priori expectation is that the learners in a learning environment, unlike in a testing environment, tend to develop their knowledge by interacting with the items rendered (and by getting feedback on their responses), and their true ability levels consequently evolve in real time. Therefore, a first step toward the goal of the adaptive learning system of optimizing the learning gain is tracing the learners' ability evolution in a fast and accurate manner.

In the context of intelligent tutoring systems (ITS), there are specialized approaches for tracing the learner's mastery of knowledge. A representative example is Bayesian knowledge tracing (BKT; Corbett and Anderson, 1994). In BKT, the learner's knowledge state is represented by a set of multiple binary latent variables that indicate mastery or non-mastery of the skills. The probability of having mastered each skill is estimated by binary measurement outcomes (correct or incorrect responses to items) and iteratively updated by using the rule of Bayes. Similar to CAT, however, the methods require a calibration on large samples using some nontrivial estimation techniques (expectation-maximization algorithm, or exhaustive search) that require high computational power (Papousek et al., 2014; Pálanek, 2016).

To that extent, an interesting alternative that can be considered for tracking the learner's ability evolution is the Elo rating system (ERS; Elo, 1978). The ERS was originally developed for calculating relative skill levels of players in chess performances, and the method also has widely been used in sport statistics for paired competitions (e.g., major league baseball). More recently, the ERS has been applied to various contexts of educational and psychological studies (e.g., Attali, 2014; Brinkhuis et al., 2015). In regard of its application to online-learning environment, the paired competition can be thought of as an interaction between the learner and the item. In general, the ERS algorithm is formulated to update the learner ability and item difficulty parameters from the Rasch model. To be specific, once a learner has responded to an item, the ERS updates the individual learner's ability level estimate that was based on his or her previous trajectory. Given the learner's current ability level, the next item is chosen by its difficulty level. A practical strength of this approach is that the method is conceptually fast and readily implementable in any software.

Several articles compared the performance of ERS with that of traditional IRT modeling to explore whether its parameter estimation is as accurate as the traditional approach. Maris and Van der Maas (2012) showed that the ability estimates updated from ERS method is highly correlated with the expected a posteriori (EAP) estimates from an IRT model when a speed-accuracy trade-off scoring rule was used. Studies also compared the performance of ERS with alternative methods for estimating item difficulties. For example, Wauters et al. (2012) compared the quality of the ERS-based item difficulty estimates with those based on maximum likelihood procedures, proportion correct, and human judgement methods, and found that the ERS provides reliable results with a sample size of 200 learners. Similarly, Pálanek (2016) provided evidence that there is a high correlation between ERS-based item difficulty estimates and joint maximum likelihood-based estimates.

Researchers (e.g., Klinkenberg et al., 2011; Savi et al., 2015; Braithwaite et al., 2016; Coomans et al., 2016; Hofman et al., 2018) also provided empirical evidence in favor of the ERS, by means of massive log data from Maths Garden, a CAP system where the learner ability and item difficulty levels are updated on the fly. Park et al. (2018) proposed a method to alleviate the cold-start in adaptive learning systems—the problem that for new learners we do not have an idea of their ability and therefore the adaptive learning environment might not perform well until the learner made a substantial number of items. The authors proposed using an explanatory IRT model based on learner-item interaction data and learner features (e.g., age, gender, or learning disability) and estimate the learners initial ability levels and their ability changes while not engaged in the learning environment.

Despite the increasing number of studies applying the ERS in adaptive learning systems, in the majority of these studies, the ERS is intended to track just a single broad ability. In contrast, monitoring multiple abilities not only forms the basis of learners' understanding of the material, but also provides direct information to educational researchers and instructors as to the areas that learners need to improve upon (Ferrini-Mundy and Schmidt, 2005). Therefore, identifying his or her progress on more fine-grained ability dimensions would imply an important advancement of the adaptive learning system, because of the sheer amount of information about the learner's learning state. Doebler et al. (2015) and Pálanek (2016) proposed an improved ERS algorithm for tracking multiple dimensions of ability. Yet, their methodological focus is still on situations where items are allowed to load on only one of the multiple ability dimensions in the answering process. More recently, Chen et al. (2018) and Tang et al. (2018) used a Markov decision process to track multiple dimensions of ability. In these studies, the learner's ability was modeled by a set of multiple binary latent variables that indicate mastery or non-mastery of the skills while a reinforcement learning approach was proposed to recommend personalized items.

In the current article, therefore, we propose to address these issues by using a multidimensional IRT (MIRT) model to track the (continuous) ability parameter estimates within ERS. The basic idea is that instead of assuming a unidimensional trait of item responses, our approach will assume that a single item may involve more than one ability parameters. Therefore, we extend the standard ERS that updates a single ability parameter based on the Rasch model, and will allow to have a simultaneous update on multiple ability parameters based on a compensatory MIRT model (“M-ERS”).

In the next section we give more details on the methodological framework of the ERS and its application to educational settings. We then propose our method (“M-ERS”) that is formulated to update multiple abilities. Next, we will evaluate the performance of our method through three simulation studies. Furthermore, the method will be demonstrated using a real application of learning data obtained from an educational platform for children's math ability development. We end with conclusions and implications.

## Elo Rating System

The ERS is originally rooted in the Bradley Terry Luce (BTL; Bradley and Terry, 1952) model, a probabilistic model that predicts the outcome of players in a type of paired competitions. Specifically, the expected outcome that one player defeats his or her opponent is formulated as follows:

(1) $$\begin{array}{l} P_{ij}=P\left(i\;defeats\;j\right)=\;\frac{\theta_{i}}{\theta_{i}+\theta_{j}}, \end{array}$$

where θ_*i*_ and θ_*j*_ represent the ratings (e.g., latent traits) of players *i* and *j*, respectively. By setting up $\theta_{i}=e^{\theta_{i}}$, Equation (1) can be transformed to a logistic function of the difference between θ_*i*_ and θ_*j*_, which comprises of the expected outcome of the ERS. That is,

(2) $$\begin{array}{l} P_{ij}=P\left(i\;defeats\;j\right)=\frac{exp\left(\theta_{i}-\theta_{j}\right)}{1+exp\left(\theta_{i}-\theta_{j}\right)} \end{array}$$

Likewise, both the BTL model and the ERS are based on the probability of winning a competition; however, the latter method is additionally intended to supply easy-to-compute updates as new outcomes are observed. In other words, the ERS takes an algorithmic heuristic to easily update the expected outcome for the next iteration, based upon the estimated latent trait (i.e., θ_*i*_ and θ_*j*_) at the current iteration. Kiraly and Qian (2017) showed that the derivative of a likelihood function for Equation (2) based on a single data point produces the following updating component for the ERS algorithm:

(3) $$\begin{array}{l} \frac{\partial l\left(\theta_{i},\theta_{j}|Y_{ij}\right)}{\partial \theta_{i}}=Y_{ij}(1-P_{ij})-\left(1-Y_{ij}\right)P_{ij}=Y_{ij}-P_{ij}, \end{array}$$

where *l*(θ_*i*_, θ_*j*_|*Y*_*ij*_) = *Y*_*ij*_ log P_ij_+(1−*Y*_*ij*_)log(1−*P*_*ij*_).

In sum, given the observations for a competition between players *i* and *j*, the estimates of θ_*i*_ and θ_*j*_ are updated simultaneously. Specifically,

(4) $$\begin{array}{l} {\hat{\theta }}_{i}=\;{\hat{\theta }}_{i}+K\;\left\{Y_{ij}-P_{ij}\right\}\text{ for a player i},\\ {\hat{\theta }}_{j}=\;{\hat{\theta }}_{j}-K\;\left\{Y_{ij}-P_{ij}\right\}\text{for a player j}. \end{array}$$

In the equation above, the term {*Y*_*ij*_ − *P*_*ij*_} can be viewed as the discrepancy between what is expected and what is observed. In fact, the ERS can be viewed as a type of the stochastic gradient descent (SGD; Robbins and Monro, 1951) algorithm where the updating rule in the system corresponds to the update of parameters along the error gradient (Pálanek, 2016). The update will be larger if the current parameter setting produces a large discrepancy. Note that *K* is a step size that defines to what extent the ability estimate can be affected by the difference between the current and expected responses for the student *p*.

## Application to Adaptive Learning Systems

In adaptive learning environments, the paired competition occurs when the learner interacts with the learning material (= item). The ERS process can be applied as follows. Consider θ_*i*(*t*)_ be an ability of a learner *i* (unidimensional continuous variable) after solving an item at measurement occasion *t*. Also, suppose *Y*_*ij*(*t*)_ be the learner *i* outcome for item *j* measured at measurement occasion *t*, where the outcome is dichotomously scored (0 = incorrect; 1 = correct answer to the item). Then the ERS for updating the ability parameter takes the following sequence:

(5) $$\begin{array}{l} {\hat{\theta }}_{i\left(t\right)}=\;{\hat{\theta }}_{i\left(t-1\right)}+K\;\left\{Y_{ij\left(t\right)}-P_{ij\left(t\right)}\right\}\text{for a learner i}\\ {\hat{\beta }}_{j\left(t\right)}=\;{\hat{\beta }}_{j\left(t-1\right)}-K\;\left\{Y_{ij\left(t\right)}-P_{ij\left(t\right)}\right\}\text{for an item j}, \end{array}$$

where ${\hat{\theta }}_{i\left(t-1\right)}$ is the ability estimate at the previous measurement time *t*−1 for the learner *i*, ${\hat{\beta }}_{j\left(t-1\right)}$ is the item difficulty estimate at the previous measurement time *t*−1 for the item *j*, and *P*_*ij*(*t*)_ is the expected response for the current measurement occasion *t*. Consequently, a learner interacting with a very difficult item risks losing a little bit of ability level in case of failure, with the possibility of gaining much greater ability level in case of success. Several studies have explored the optimal step size *K* for the ERS for student modeling. Wauters et al. (2012) suggested using a constant step size, *K* = 0.4 in the context of educational data. On the other hand, other studies (e.g., Glickman, 1999; Klinkenberg et al., 2011; Papousek et al., 2014; NiŽnan et al., 2015) proposed that the step size needs to decrease as a function of a total number of item answered and therefore the system gains more information about the learner's true ability level.

In Equation (5), it is possible that the outcome *Y*_*ij*(*t*)_ can be scored by considering whether the learner completed the item within the allotted limit. Maris and Van der Maas (2012) derived a scoring rule that accounts for response time and accuracy, and applied it within ERS. While the ERS can be used to gradually obtain reliable estimates of both student's abilities and item difficulties, adaptive item sequencing can be more efficient if we could start from a pre-calibrated item bank, including information on item difficulty and possibly other characteristics of items, and from which items with undesired characteristics are excluded (van Groen et al., 2014). In this case, ${\hat{\beta }}_{j\left(t\right)}$ in Equation (5) needs not be updated.

## Multidimensional Extension of the ERS (M-ERS)

In this section we propose an extended version of the ERS that enables the system to track multidimensional abilities in real time. Specifically, the proposed algorithm can handle two types of dimensional structures in the item bank–(a) “within-item dimensionality” where a single item can be associated with more than one task ability; as well as (b) “between-item dimensionality” where a set of items is associated with multiple abilities, while each item measures only one of those abilities.

Suppose an adaptive learning environment contains an item bank that is designed to measure a total of *M*-dimensional abilities i.e., $\theta_{i}={\left(\theta_{i1},\;\ldots ,\theta_{iM}\right)}^{{}^{\prime }}$for a student *i*. The multidimensional dichotomous logistic model (Reckase, 1985) that describes the probability of a correct answer to item *j* can be formulated by either conjunctive or compensatory assumptions about how the latent abilities are combined. In a conjunctive model assumption, it is assumed that the learner should have each of the relevant abilities in order to answer an item correctly. The probability of a correct response therefore is a joint product of the inverse logit function of the difference between each of the abilities and the corresponding item difficulty:

(6) $$\begin{array}{l} P_{ij}=P\left(Y_{ij}=1\right)=\prod_{m=1}^{M}\frac{exp\left(\alpha_{jm}\left[\theta_{im}-\beta_{j}\right]\right)}{1+exp\left(\alpha_{jm}\left[\theta_{im}-\beta_{j}\right]\right)}, \end{array}$$

where *P* (*Y*_*ij*_ = 1) indicates the probability of a correct answer, θ_*im*_ is the *m*th ability parameter of the learner *i* (*m* = 1, …, *M*), α_*jm*_ is the item discrimination of the item *j* corresponding to *m*th ability dimension, and β_*j*_ denotes the overall difficulty level of the item *j*. On the other hand, in a compensatory model, on the other hand, it is assumed that the lack of one ability can be compensated by greater level of another ability, as follows:

(7) $$\begin{array}{l} P_{ij}=P\left(Y_{ij}=1\right)=\frac{exp\left(\sum_{m=1}^{M}\alpha_{jm}\theta_{im}-\beta_{j}\right)}{1+exp\left(\sum_{m=1}^{M}\alpha_{jm}\theta_{im}-\beta_{j}\right)}. \end{array}$$

The difference between the observed and the expected performance *P*_*ij*_ based on the multidimensional IRT models is used to update the ability parameters after each item response. Specifically, the *P*_*ij*_ within ERS for the *m*-th ability for person *i* on measurement occasion *t* is updated as follows:

(8) $$\begin{array}{c} {\hat{\theta }}_{im\left(t\right)}=\;{\hat{\theta }}_{im\left(t-1\right)}+D_{m\left(t\right)}\;K\;\left\{Y_{ij\left(t\right)}-P_{ij\left(t\right)}\right\}\\ {\hat{\beta }}_{j\left(t\right)}=\;{\hat{\beta }}_{j\left(t-1\right)}-D_{m\left(t\right)}\;K\;\left\{Y_{ij\left(t\right)}-P_{ij\left(t\right)}\;\right\}, \end{array}$$

where *D*_*m*(*t*)_ is a weight to specify whether the *m*th ability is indicated by the item given at *t*-th step. For the ability that is indicated by the item, *D*_*m*(*t*)_ equals 1. For the ability that is not indicated by the item, the weight takes values between zero and one.

## Simulation Study

To explore the performance of the M-ERS method in terms of estimating the real-time evolution of multidimensional ability parameters for individual learners, we apply the method to data generated under a variety of conditions. In accordance with our research questions, the simulation study consists of three parts. In Study 1, we examine the result of a standard ERS that naively assumes the unidimensionality of ability parameter, where in fact data involve a multidimensional ability. In Study 2, we explore the performance of the M-ERS in relation to the total number of items answered. In Study 3, we investigate the performance of the modified M-ERS in which both response time and accuracy data are incorporated.

### Item Bank

Following the literature on the MIRT (e.g., Adams et al., 1997; Hartig and Höhler, 2008), we consider two loading structures to determine patterns of the multidimensionality. Specifically, two types of item banks are created– (a) when items are allowed to load on more than a single ability dimension (“Item bank 1”); and (b) when items are allowed to load on only one of the multiple ability dimensions (“Item bank 2”). Each of them includes 200 operational items, measuring a total of three dimensions. Item bank 1 consists of a primary dimension θ_1_ indicated by all the items, and two auxiliary dimensions, θ_2_ and θ_3_, indicated by 35% of items and the 25% of the remaining items. In Item bank 2, each item involves only one out of the three dimensions. Specifically, 40% of items involves the 1st dimension, 35% of them involves the 2nd dimension, and the remaining 25% of them involves the 3rd dimension (as an illustration, Table 1 gives a sample of 15 items from two item banks). Based on each item bank, data are generated under a compensatory IRT model with difficulty and discrimination parameters, mimicking realistic test items:

(9) $$\begin{array}{l} P_{ij}=P\left(Y_{ij}=1\right)=\frac{\exp\left(\sum_{m=1}^{3}\alpha_{mj}\theta_{jm}-\beta_{j}\right)}{1+\exp\left(\sum_{m=1}^{3}\alpha_{mj}\theta_{jm}-\beta_{j}\right)}, \end{array}$$

where the generating parameter values for the difficulty and the slope parameters are drawn from β_*j*_ ~ *N*(0, 1) and α_*mj*_ ~ *U*(0.5, 2), where *j* = 1,…, 200 (items) and *m* = 1, 2, 3 (dimensions of ability).

**Table 1:** Patterns of multidimensionality (a sample of 15 items from two item banks).

**Item ID**	**β_j_**	**Item bank 1**			**Item bank 2**		
		**θ_1_**	**θ_2_**	**θ_3_**	**θ_1_**	**θ_2_**	**θ_3_**
1	−2.534	1	.	.	1	.	.
2	−2.21	1	.	.	1	.	.
3	1.326	1	.	.	1	.	.
4	0.253	1	.	.	1	.	.
5	1.275	1	.	.	1	.	.
6	0.089	1	.	.	1	.	.
7	−0.001	1	1	.	.	1	.
8	−1.256	1	1	.	.	1	.
9	2.242	1	1	.	.	1	.
10	−1.556	1	1	.	.	1	.
11	2.213	1	1	.	.	1	.
12	−3.3	1	.	1	.	.	1
13	0.753	1	.	1	.	.	1
14	−2.246	1	.	1	.	.	1
15	−1.156	1	.	1	.	.	1

“*1” indicates that the item loads on the dimension*.

### Persons

A total of *n* = 250 learners are considered in the simulation studies. The population distribution of ability parameters is taken to be *N*(μ, **Σ**), where μ**=**(1, 1, 1)′. In **Σ**, all variances are equal to 1 and the three bivariate correlations are equal: dimensions were independent ($\rho_{mm^{\prime }}\;=\;$0.0), weakly correlated ($\rho_{mm^{\prime }}\;=\;$0.2), or moderately correlated ($\rho_{mm^{\prime }}\;=\;$0.5).

A total of 6 data sets were generated by following 6 scenarios (2 patterns of dimensionality × 3 correlations among ability dimensions). In each condition, the M-ERS method will be used to update the three ability parameter estimates for each learner as he or she attempts on a sequence of items. We assume that each learner is assigned a sequence of 200 items that are randomly selected. That is, item sequences are varied across learners. In M-ERS, in Equation (7), the expected response is estimated by using a compensatory IRT model with a constraint that the slope parameters α_*mj*_'s are equal to 1 for simplicity (the inclusion of different loadings is a logical further extension). That is,

$$\begin{array}{l} P\left(Y_{ij\left(t\right)}=1\right)=\frac{\exp\left(\sum_{m=1}^{3}\theta_{jm}-\beta_{j}\right)}{1+\exp\left(\sum_{m=1}^{3}\theta_{jm}-\beta_{j}\;\right)}. \end{array}$$

In the equation, we use step size K that linearly decreases as a function of a total number of items answered between the maximum value of 0.4 and the minimum value of 0.1. Note that we assume that item difficulty parameters are considered as known (based on a calibration study), and the difficulty estimates therefore will not be further updated within the M-ERS.

## Study 1: Prediction Accuracy of a Standard ERS and M-ERS

In the first study, we explore the extent to which the unidimensional ability assumption embedded in a standard ERS has an impact on the prediction accuracy (in terms of the learners' future responses), when the truth is that the responses to the learning items involve three-dimensional ability parameters. As seen in Equation (8), predictions of the responses of learners can be achieved by using the ERS algorithm based on the known item parameters and the learner ability estimate(s) predicted by the preceding step. The prediction accuracy is calculated by classifying the expected response (= *P*_*ij*(*t*)_) with a certain cut-point into the observed response (= *Y*_*ij*(*t*)_) on measurement occasion *t*. To evaluate the quality of predictions, we use a Receiver Operating Characteristic curve (ROC). The ROC curve represents the relation between true positive rates [ = TP/(TP + FN)] and false positive rates [FP/(FP + FN)] at various probability cut-off points. In case of totally random predictions, the Area Under Receiver Operating Characteristic curve (AUROC) is approximately equal to 0.5.

**Figure 1:**
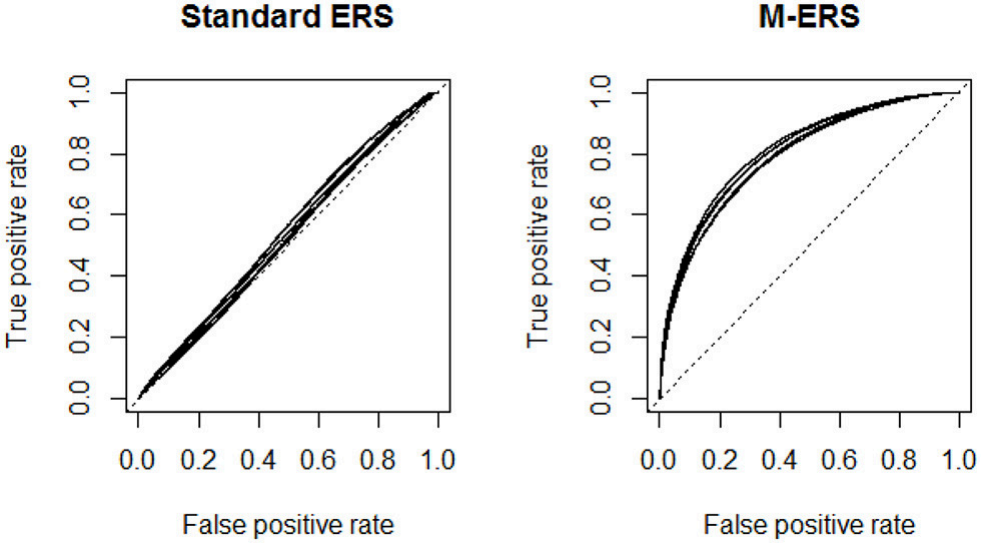
Receiver operating characteristic (ROC) curves for a standard ERS and M-ERS. *Note*. Each panel includes six ROC curves representing a total of 6 simulation conditions (2 patterns of dimensionality × 3 correlations among ability parameters).

Figure 1 visualizes the ROC curves, comparing the performances of a standard ERS and the M-ERS. Each panel in the figure includes 6 curves, representing a combination of simulation conditions (2 patterns of dimensionality × 3 correlations among ability parameters). The x-axis and y-axis indicate the false positive rate and the true positive rate. Note that the best possible prediction method would yield a point in the upper left corner or coordinate (0, 1) of the ROC space, representing 100% sensitivity and specificity. Therefore, results from the two panels suggest that M-ERS outperforms the standard ERS in all six simulated scenarios. Table 2 summarizes the area under the ROC curves (AUROC). In case of M-ERS, the AUROCs are much higher than the expected values using random predictions (i.e., 0.5 for AUROC). It is seen that the AUROCs for M-ERS are 0.8088 and 0.7881 for Item banks 1 and 2, respectively. However, the standard ERS generally reveals only performs marginally better than the random predictions (i.e., 0.5299 for Item bank 1 and 0.5345 for Item bank 2).

**Table 2:** Area Under ROC curve (AUROC) for a standard ERS and M-ERS.

	**Item bank 1**		**Item bank 2**	
	**Standard ERS**	**M-ERS**	**Standard ERS**	**M-ERS**
ρ = 0.0	0.5197	0.8038	0.5216	0.7850
ρ = 0.2	0.5204	0.8070	0.5324	0.7900
ρ = 0.5	0.5496	0.8157	0.5494	0.7893
Average	0.5299	0.8088	0.5345	0.7881

## Study 2: Ability parameter estimation as a function of number of items answered

In the second study, we investigate the performance of M-ERS as a function of the total number of items answered. We also examine the effect of different simulation conditions (i.e., dimensionality patterns and correlations among true three ability parameters) on the ability parameter estimation. To evaluate the quality of the ability estimation, the estimated ability parameters are summarized by mean squared error (MSE). In particular, the differences between the true and estimated abilities at measurement occasion *t* are squared, and averaged over the entire sample size of new students. That is, for the learner *i* at the measurement occasion *t*:

(10) $$\begin{array}{l} MSE\left({\hat{\theta }}_{m\left(t\right)}\right)=\frac{\sum_{i=1}^{N}{\left({\hat{\theta }}_{im\left(t\right)}-\theta_{im\left(t\right)}\right)}^{2}}{N} \end{array}$$

**Figure 2:**
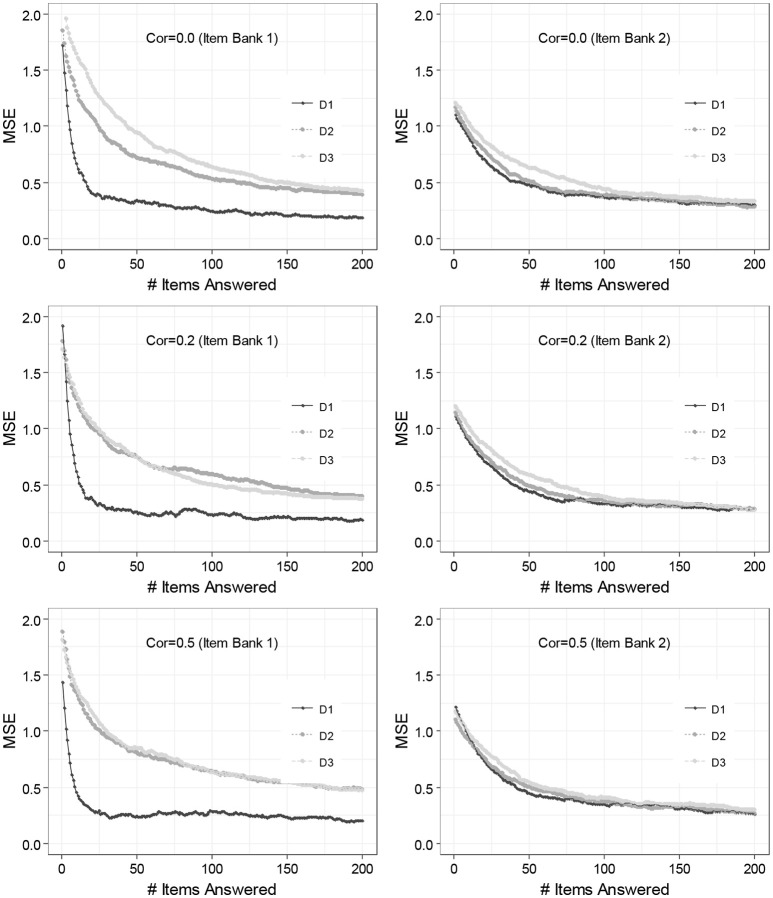
Result of 3-dimensional ability estimation across the number of items answered. Cor, correlations between dimensions; D1, 1st dimension; D2, 2nd dimension; D3, 3rd dimension.

Figure 2 includes line plots demonstrating the performances of the M-ERS for two different patterns of dimensionality. Each panel in the figure represents a different simulation condition with the patterns of dimensionality (Item banks 1 and 2) and the true correlations among ability parameters ($\rho_{mm^{\prime }}=$ 0.0, 0.2, and 0.5), with a total number of items answered being on the x-axis and the MSEs on the y-axis. The three lines in each panel comprise the squared difference between the ${\hat{\theta }}_{im\left(t\right)}$ and θ_*im*(*t*)_ averaged across *n* = 250 individual learners for the three dimensions (“D1,” “D2,” and “D3” in the legend). Remind that the three panels on the left-hand side summarize the performance of the M-ERS method for Item bank 1 that exhibits one primary dimensions plus two auxiliary dimensions (Item bank 1). The other three on the right-hand side are based on the data where each item involves only a single dimension. See the section of “Study Design” for more details.

Overall, results suggest that that the MSEs tend to decrease as the total number of items answered increases. The finding is common to all three dimensions (“D1,” “D2,” and “D3”), but the speed of decrease varies by the number of items the ability dimension involves. For Item bank 1 (column left), it is seen that MSE for the 1st dimension (on which all items load) reveals a dramatic decrease while the first 20 items are answered (to around 0.2). Similarly, though more gradually, MSEs for 2nd and 3rd dimensions (on which only 35 and 25% of the items load) also tend to decrease. In these two auxiliary dimensions, however, the MSE does not reach 0.2, even up to 200 items.

For Item bank 2 (column right), on the other hand, the degrees of the decreasing trends are extremely similar among the three dimensions. This can be explained by the fact that the three dimensions involve a similar amount of items i.e., 40, 35, and 25% of items load on the D1, D2, and D3, respectively. For moderate scenarios, in particular, the difference becomes extremely tiny. It is seen that the true correlations among ability parameters do not have an impact on MSEs across any measurement occasions.

**Figure 3:**
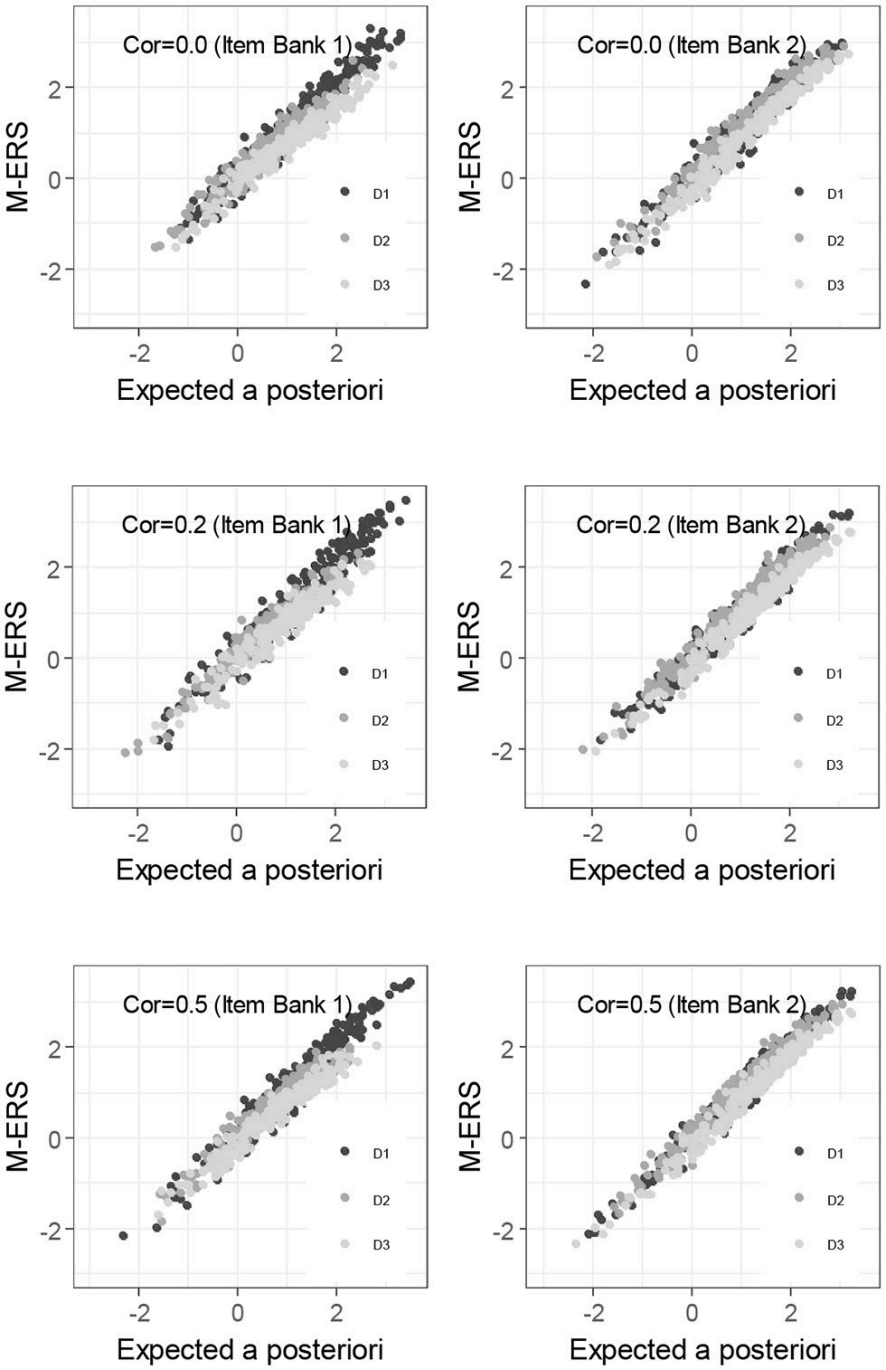
Relations between EAP and M-ERS estimates.

As an alternative way to check the performance of M-ERS, Figure 3 compares the ability estimates of 250 learners after 200 Elo-updates with the expected a posteriori (EAP) ability estimates obtained by fitting a compensatory IRT approach. Overall, ability estimates from EAP and M-ERS are highly correlated, regardless of any simulation conditions and the ability dimensions. The correlation coefficients (3 dimensions × 2 item banks × 3 correlation among true abilities) range from 0.967 to 0.990. Note that the EAP estimates are the results of an analysis that requires responses of many persons on many items, and is computing-intensive and therefore cannot be used on the fly. Therefore, the EAP estimates are used here as a benchmark, but they cannot be considered as a viable alternative for the ERS approach.

## Study 3: M-ERS for Speed-Accuracy Tradeoff

There have been an increasing number of studies (e.g., Tuerlinckx and De Boeck, 2005; van der Linden, 2007; De Boeck et al., 2017) that account for response time as well as response accuracy in order to model the ability parameter. Of several statistical and psychological approaches to the response time modeling (van der Linden, 2009), one of the promising methods is to model response time and accuracy from the measurement perspective by two-step procedures; specifically, (a) setting up a scoring rule and (b) fitting a proper statistical model that conforms scores of the type. Klinkenberg et al. (2011) showed that the ERS method outperforms a standard CAT method (specifically, Eggen and Verschoor, 2006) when the speed-accuracy trade-off scores [so called high speed high stake (HSHS)] and the corresponding model were used. We do not know studies that model multidimensional ability trajectories based on both response time and accuracy within ERS. Therefore, we aim to explore the incorporation of the HSHS scoring rule in the proposed M-ERS method.

According to the HSHS scoring rule, the observed scores can be calculated as follows: *S*_*ij*_ = (2*Y*_*ij*_ − 1)(*d* − *T*_*ij*_), where *Y*_*ij*_ is an accuracy for the learner *i*'s response to the item *j*, *d* is a time limit, and *T*_*ij*_ is an time spent for the learner *i* until answering the item *j*. In this expression the residual time i.e., (*d* − *T*_*ij*_) can compensate or penalize for the learner, corresponding to the learner's accuracy to the item. In particular, for a correct response (i.e., *Y*_*ij*_ = 1), the learner will gain the residual time as a score. Similarly, for an incorrect response (i.e., *Y*_*ij*_ = 0), the score will be reduced by the same amount. In current study, the maximum time given each item (= *d*) is restricted to be 1, so the residual time simply reflects the proportion of time left. Such a scoring scheme is especially useful to control for the case that the learner guesses instantaneously guess the given item (a quick incorrect answer). The expectation of the trade-off score, *E*(*S*_*ij*_), for an item that is based on three abilities can naturally be extended from a unidimensional version in Maris and Van der Maas (2012). That is,

(11) $$\begin{array}{l} E\left(S_{ij}\right)=\;\frac{\exp\left(2\left(\sum_{m=1}^{3}\theta_{im}-\beta_{j}\right)\right)+1}{\exp\left(2\left(\sum_{m=1}^{3}\theta_{im}-\beta_{j}\right)\right)-1}-\frac{1}{\left(\sum_{m=1}^{3}\theta_{im}-\beta_{j}\right)}, \end{array}$$

where *m* = 1, 2, 3 (abilities). Specifically, the *E*(*S*_*ij*_) in Equation (10) provides the expected HSHS score for learner *i*'s to solve the item *j*.

We conduct a simulation study to compare this approach with the M-ERS method based on the accuracy data only. The entire data-generating process follows what is described in the section “Study Design.” The response time data is generated for each learner who solves each item, using a formula for expected response time from Maris and Van der Maas (2012), where the time limit for each item is consistently set at 1-min. Like in studies 1 and 2, the simulation conditions are combinations of patterns of dimensionality (Item banks 1 and 2) and the true correlations among ability parameters ($\rho_{mm^{\prime }}=$ 0.0, 0.2, and 0.5).

Table 3 comprises two tables that compare the performance of two M-ERS method when only accuracy data are used (“Accuracy”) or both speed and accuracy data (“Speed-Accuracy”) are used. Overall results suggest that M-ERS for speed-accuracy data shows smaller MSE, regardless of any simulation conditions.

**Table 3:** Comparing IRT-based ERS algorithms for correctness vs. trade-off score (correctness and speediness combined).

		**ρ** **=** **0.0**		**ρ** **=** **0.2**		**ρ** **=** **0.5**	
		**|Bias|**	**MSE**	**|Bias|**	**MSE**	**|Bias|**	**MSE**
**ITEM BANK 1**							
${\hat{\theta }}_{1}$	Accuracy	0.009	0.300	0.008	0.286	0.009	0.289
	Speed-accuracy	0.008	0.245	0.008	0.232	0.007	0.213
${\hat{\theta }}_{2}$	Accuracy	0.010	0.364	0.009	0.363	0.010	0.382
	Speed-accuracy	0.009	0.306	0.009	0.298	0.009	0.307
${\hat{\theta }}_{3}$	Accuracy	0.010	0.400	0.010	0.395	0.010	0.392
	Speed-accuracy	0.009	0.337	0.009	0.336	0.009	0.332
**ITEM BANK 2**							
${\hat{\theta }}_{1}$	Accuracy	0.008	0.291	0.008	0.294	0.008	0.290
	Speed-accuracy	0.008	0.245	0.008	0.251	0.008	0.252
${\hat{\theta }}_{2}$	Accuracy	0.008	0.299	0.008	0.290	0.008	0.291
	Speed-accuracy	0.008	0.260	0.008	0.245	0.008	0.252
${\hat{\theta }}_{3}$	Accuracy	0.009	0.337	0.009	0.353	0.009	0.315
	Speed-accuracy	0.008	0.299	0.009	0.310	0.008	0.278

*|Bias|, absolute value of the bias averaged over learners and items; MSE, a mean squared error averaged over learners and items*.

## Real Data Analysis

### Description

For illustrative purposes, we used a dataset collected from a web-based learning platform, “Number Sense” (Linsen et al., 2016) developed by KU Leuven, Belgium. It was designed as an item-based e-learning environment for 6- to 8-year-old children and includes approximate number discrimination tasks, symbolic comparison tasks, and symbolic and non-symbolic number line estimation tasks. In particular, current data were collected between Fall 2017 and Spring 2018, during one school year. It includes data from 299 students' responses to 330 items in total. Among the items, 168 of them are designed to measure (a) comparison ability and the remaining 162 items are designed to measure (b) number line estimation ability. There were no items that require both. All responses to the items are scored for accuracy i.e., the binary scale (correct/incorrect). Current log data do not include response times.

For the purpose of obtaining item parameters, data from 200 out of 299 students were used to fit a MIRT formula i.e., $P\left(Y_{ij}=1\right)=\frac{\exp\left(\theta_{j1}+\theta_{j2}-\beta_{j}\right)}{1+\exp\left(\theta_{j1}+\theta_{j2}-\beta_{j}\right)}$, where θ_*i*1_ and θ_*i*2_ reflect abilities in relation to the comparison and number line estimation, respectively. The remaining 99 students were used to illustrate the Elo algorithm. For the estimation procedure with this training set, the MCMC algorithm is implemented with R 3.3.3 (R Core Team, 2013). More specifically, JAGS (Plummer, 2015) was implemented by an R package “R2jags” (Su and Yajima, 2015) that provides wrapper functions for the Bayesian analysis program. For each analysis with the JAGS, four chains were run, and each ran for 10,000 iterations. We used a thinning parameter of four and used the first half as burn-in. (Gelman and Rubin's, 1995) statistics are used for a convergence diagnostic. Results of the Bayesian inference show that the posterior predictive mean for the correlation between the comparison and number line estimation abilities (i.e., θ_*i*1_ and θ_*i*2_) was approximately $\hat{\rho }=$ 0.13. The posterior predictive means for item discriminations and difficulties (i.e., ${\hat{\alpha }}_{mj}$ and ${\hat{\beta }}_{j}$) were used as known item parameters, and where therefore not updated within the Elo algorithm.

### Results

**Figure 4:**
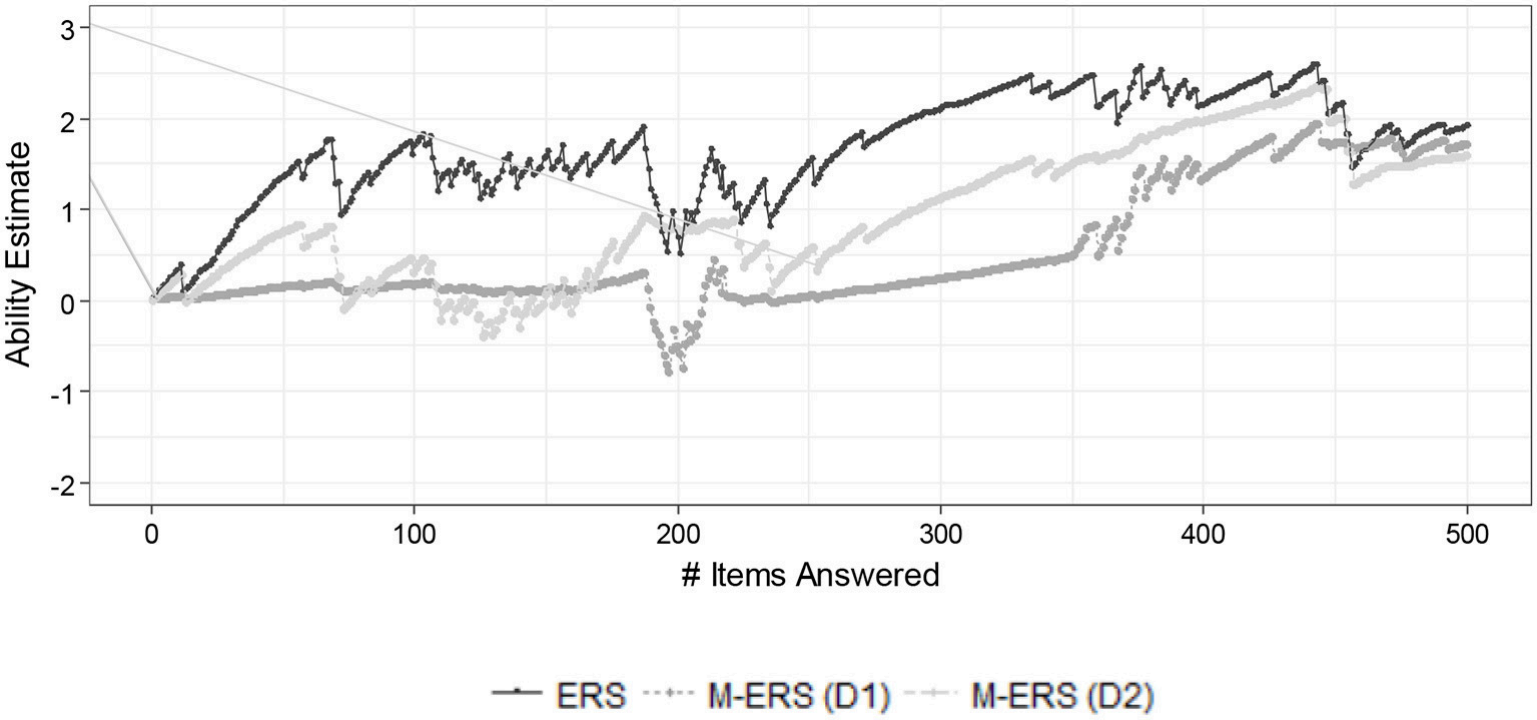
Example of ability estimates for a student by standard ERS and M-ERS.

Figure 4 shows the resulting ability trajectory of two randomly chosen students by fitting a standard ERS and the M-ERS. As in the simulation result, the figure presents the impact of assuming unidimensionality or multidimensionality of the ability parameter. It is noticeable that the ability estimates obtained by a standard ERS are in general greater than the two-dimensional ability estimates from the M-ERS. Based on the similarity of the Number Sense data to the data we generated in the simulation and the results we found there, we can suspect that the unidimensional ERS ability estimates for the Number Sense items are biased (upward), and the M-ERS has removed the bias. That implies that ignoring the multidimensional data structure may cause considerable bias in ability trajectory estimation in the learning environment, and therefore in a suboptimal adaptivity of the learning environment. After a longer sequence of items in the session, however, it is shown that the gaps among four approaches tend to be negligible.

## Conclusion

In this paper, we have proposed an MIRT-based ERS method to address a dynamic estimation of the learner's progress in an adaptive practice environment where the learning items exhibit a multidimensional ability criteria. The model combines the idea of using a compensatory MIRT model to predict the learner performance with a fast and heuristic algorithm for tracking his or her irregular trend of ability parameters through the ERS.

First, we have shown that there occurs a considerable error in terms of updating the ability changes, when a unidimensional IRT is used in ERS when the truth is that there is a multidimensionality in a set of items. We have shown that the error in estimating the ability parameters can be alleviated with the compensatory IRT in ERS. Second, we have shown that at the initial step of learning, the error of ability estimates are bigger where each individual item involves more than one dimensions, as compared to the case where the item purely involves one of the multiple dimensions. However, we have found that the error has been noticeably reduced as more items are rendered. Third, we have extended the M-ERS method for the trade-off scores between response time and accuracy. Results show that bias and MSE of ability estimates are smaller when the HSHS (i.e., the speed-accuracy trade-off scoring that gives penalty for guessing) was incorporated in M-ERS than using the accuracy data only.

We believe that our approach offers the possibility to improve adaptivity when applied in an adaptive environment. In our simulation study, the items were chosen randomly across measurement occasions for each student, but it is also possible to administer items that optimize the item selection criteria. For example, the item can be chosen such that its difficulty level is as close as possible to the learner's current ability (e.g., 50% chance of answering correctly). In the ERS based on the IRT formula, such an item selection strategy can be flexibly adjusted to avoid too easy (e.g., 90% chance of answering correctly) or too hard (e.g., 25% chance of answering correctly) items to individual learners. That means that the ERS can provide a flexible item sequencing tool for adaptivity in which a series of items are updated in real time based on their ability or knowledge levels (Wauters et al., 2010).

Another idea that may arise when considering to deal with ability estimation in ERS is to handle the cold-start problem i.e., the system does not know a new learner's ability level in the beginning of learning stage, when the new learner comes into the e-learning system for the first time. The cold start problem may also occur when a learner leave the e-learning system for a while and return (i.e., between-session period) because external effects could lead to the ability level change. Finally, the current simulation study shows a few limitations. For instance, the true ability was assumed to be constant over time, although it tends to evolve in learning environments. Including a time trend can add additional challenges, such as the determination of a step size that is large enough to keep track of the evolving ability but not too large in order to avoid very instable ability estimates.

Nevertheless, we believe the results of current study provide valuable information about how to efficiently follow up estimate multidimensional ability changes in the e-learning environments in order to alleviate concerns about the ERS and catalyze the usefulness of the e-learning system in educational settings.

## Data Availability

The datasets generated for this study are available on request to the corresponding author.

## Author Contributions

JP developed the core ideas of the manuscript, the method, the design of the simulation study, as well as the real data analysis. WV supervised the entire work and gave feedback. FC and HvdM gave feedback on the results and the writing and contributed to the final manuscript.

### Conflict of Interest Statement

The authors declare that the research was conducted in the absence of any commercial or financial relationships that could be construed as a potential conflict of interest.
